# Suitability of NB-IoT for Indoor Industrial Environment: A Survey and Insights

**DOI:** 10.3390/s21165284

**Published:** 2021-08-05

**Authors:** Muhammad Dangana, Shuja Ansari, Qammer H. Abbasi, Sajjad Hussain, Muhammad Ali Imran

**Affiliations:** James Watt School of Engineering, University of Glasgow, Glasgow G12 8QQ, UK; Shuja.Ansari@glasgow.ac.uk (S.A.); Qammer.Abbasi@glasgow.ac.uk (Q.H.A.); Sajjad.Hussain@glasgow.ac.uk (S.H.); Muhammad.Imran@glasgow.ac.uk (M.A.I.)

**Keywords:** Narrow-Band IoT, Internet of Things, Industrial IoT, wireless communication, latency, self-organizing network (SON), edge computing, long term evolution (LTE)

## Abstract

The Internet of Things (IoT) and its applications in industrial settings are set to bring in the fourth industrial revolution. The industrial environment consisting of high profile manufacturing plants and a variety of equipment is inherently characterized by high reflectiveness, causing significant multi-path components that affect the propagation of wireless communications—a challenge among others that needs to be resolved. This paper provides a detailed insight into Narrow-Band IoT (NB-IoT), Industrial IoT (IIoT), and Wireless Sensor Networks (WSN) within the context of indoor industrial environments. It presents the applications of NB-IoT for industrial settings, such as the challenges associated with these applications. Furthermore, future research directions were put forth in the areas of NB-IoT network management using self-organizing network (SON) technology, edge computing for scalability enhancement, security in NB-IoT generated data, and proposing a suitable propagation model for reliable wireless communications.

## 1. Introduction

The development of ad hoc Wireless Sensor Networks (WSN) contributes to the paradigm of the Internet of Things (IoT). Many wireless technologies have emerged with this paradigm; among these are the Low Power Wide Area Network (LPWAN) such as Narrow-Band IoT (NB-IoT), Long Term Evolution for Machines (LTE-M), Long Range Wide Area Network (LoRaWAN), SigFox, etc. Since IoT involves the connectivity of massive objects to the internet for information or data gathering, monitoring, and control, a highly reliable platform is required. As such, the focus has been on the requirements that will establish this reliable connectivity. Wireless technologies are therefore geared towards meeting these requirements among which include: low power consumption, to enable longer duration of connectivity and energy savings; coverage range, for extended object reachability; reliability, for error-free data transmission and network availability; security, for data confidentiality, integrity, and availability; latency, for real-time data delivery.

The standardization of NB-IoT technology by the Third Generation Partnership Project (3GPP) in 2016 [1] has empowered NB-IoT with features that have enabled it to be suitable for wireless data communication. The expanded Discontinuous Reception (eDRx) and Power Saving Mode (PSM) techniques allow NB-IoT to implement its low power consumption scheme. For a more extended coverage range, the technology employs the re-transmission and low-frequency modulation mechanisms. For a low latency sensitivity level, 3GPP has prescribed a tolerable latency level of 10s. The superimposition of NB-IoT on Long Term Evolution (LTE) provides the support and reliability needed for its network availability.

These 3GPP standardization features have increased the presence of NB-IoT modules in the forecast of the IoT device market share by 2030 [2]. Many wireless vendors have entered into the business of IoT manufacturing. Since NB-IoT is integrated into the LTE infrastructure, this has resulted in its application in numerous industrial sectors, such as agriculture, transportation, automobile factories, logistics and manufacturing, while increasing the data transmission over LTE infrastructure. However, the structural settings of these industries differ and are comparatively unique. The uniqueness of these industries is characterized by heavy machinery and in some cases with metals that are highly reflective to wireless signals. These forms of settings present a harsh condition for wireless communications [3].

The deployment of NB-IoT in indoor industrial environments is faced with these harsh channel conditions, and as such, a more appropriate wireless propagation model is needed to describe the wireless system of NB-IoT communication in an indoor industrial environment. Attempts have been made to characterize the wireless propagation of narrow-band in industrial settings [3,4]. Reference [3] performed physical measurements of wood and metal processing factories for large-scale and temporal fading at frequencies of 900, 2400, and 5200 MHz. The authors identified and classified the features of the industrial environments that affect the large-scale and temporal fading. Of interest is the presentation made by [4] on its comparative analysis of indoor industrial environment propagation models with a generic representation of path-loss for 900 MHz.

This paper presents a survey on NB-IoT, and as a future direction, some research areas are discussed in a bid to bring forth an appropriate representation of the NB-IoT network. This is to aid in designing suitable wireless communication systems that can be used in developing a reliable scheme for the measurement of an acceptable threshold for NB-IoT wireless performance in industries, measuring the tolerable latency sensitivity level as described in 3GPP, and analyzing the effect of the propagation model on power consumption rates in NB-IoT.

**Figure 1:**
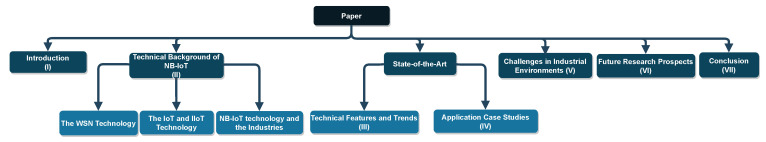
A structural description of the paper layout.

After the introduction section, in this paper, Section 2 presents the technical background on NB-IoT with brief explanations on WSN, IoT, and IIoT; this is to clarify their relations and impact on NB-IoT. Other topics presented include the standardization and technology of NB-IoT. As shown in the organizational structure of the research article in Figure 1, under the state-of-the-art, Section 3.1 discusses the developmental trends and technical features of NB-IoT, WSN, and IoT. Section 3.2 discusses the industrial application case studies of NB-IoT. The challenges posed by the industrial environments on these technologies are presented in Section 3.3. Table 1 also shows a summary of selected state-of-the-art articles. Further research directions and conclusions are presented in Section 4 and Section 5, respectively.

### Research Methodology

**Figure 2:**
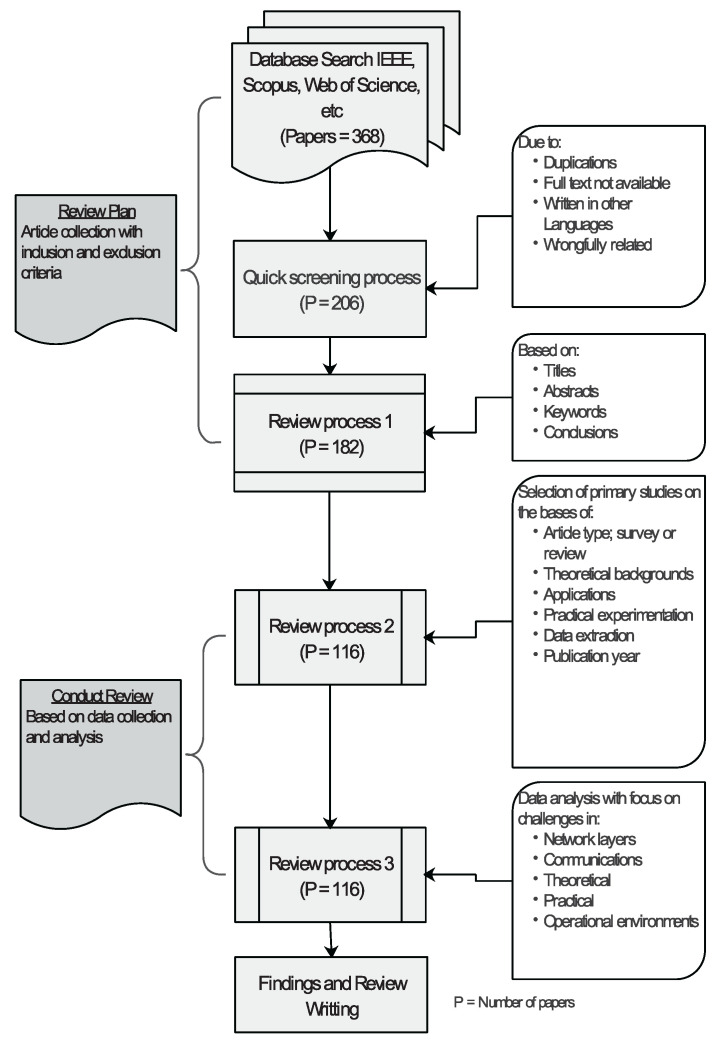
Data extraction procedure.

Among the contributions of this paper are, firstly, to review the state-of-the-art research in the field of NB-IoT technology and its applications in industrial environments. To review other IoT technologies (WSN, IoT, and IIoT) upon which NB-IoT is established. This is aimed at understanding the relationships and impact of these technologies with NB-IoT, especially in terms of technical features, applications, and challenges in industrial environments. Lastly, to highlight the challenges and future directions of wireless communication of NB-IoT in industries. This paper reviewed research carried out in recent years in the field of Industrial Internet of Things (IIoT) application with some notes on earlier contributions. The process of this review involved a thorough checking of the significant contributions made by journals, articles, conference papers, and report papers. The scholarly databases were systematically queried to extract important information from the literature identified. A total of 368 papers were pulled out of the databases, and 116 papers were selected and comprehensively read, analyzed, and categorized based on their areas of review. Figure 2 depicts the paper extraction procedures. The first process screened out or excluded papers that were in duplicates, non-English written, and had text wrongfully categorized concerning the area of review interest. The review process began with the inclusion of papers on the basis of titles, abstracts, keywords, and conclusions. A total number of 116 papers were finally selected by article type (survey or review, theoretical, practical, etc.). Data were also extracted at this stage to include both theoretical and experimental contents. The third process of the review focused on the challenges that are related to the communication characteristics as supported by the network layers.

## 2. Technical Background of NB-IoT

The evolution of the Internet of Things (IoT) has occurred in recent years, with a forecast on IoT device connectivity running into billions in 2021 [2]. The standardization and careful research into technology has also led to meaningful progress. This section, therefore, briefly describes some of the features of WSN, IoT, Industrial IoT (IIoT), and NB-IoT, as well as their technical challenges.

### 2.1. The Wireless Sensor Networks (WSN) Technology

WSN is a set of sensing nodes that are arranged in a manner that allows them to function together to monitor or read some physical quantity (such as temperature, humidity, pressure, etc.) and communicate to a central point or device wirelessly. The full structure of WSN comprises the sensors (nodes), the communication technique, and the nature of their inter-connectivity. This implies that WSN has the capabilities of sensing, processing, storing, and communicating with its data destination through the wireless medium.

#### 2.1.1. Structure of a Typical Sensor Node

**Figure 3:**
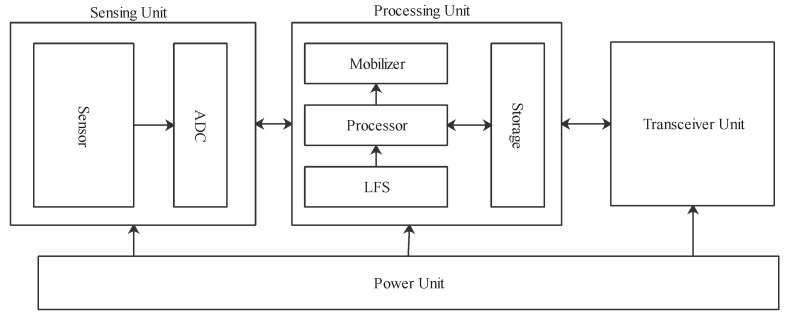
A detailed representation of the four units of an IoT node.

To understand the WSN technology, a study of a typical sensor node is important. Figure 3 shows the four major units of a node: power, sensing, processing, and transceiver units. The power unit provides the energy required by other units in the node. This power source in most cases is provided by a battery. The sensing unit monitors the physical quantities that are presented in analog form or signals. With the aid of an Analog-to-Digital converter (ADC), the analog signal is converted to a digital signal. Both the sensor and ADC form the sensing unit. The digital signal is then processed by a micro-controller or processor. The processed data are stored temporarily in the storage section of the processing unit. The transceiver unit enables the node to connect with other nodes or the gateway in the network [42].

#### 2.1.2. WSN Network Architecture

**Figure 4:**
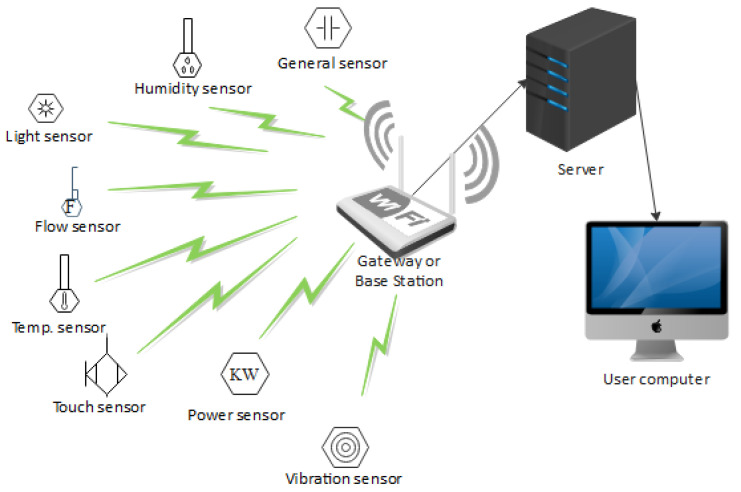
A WSN network architecture showing data sources, aggregation, storage, and control points.

The network architecture of WSN is considered to be of two types: structured and unstructured [43]. A structured WSN network design provides for a well-mannered approach to its planning and distribution of the nodes. With this type of deployment, the network maintenance rate is minimal, which leads to a low management expenditure as well. While in an unstructured network, the nodes’ distribution and placements are scattered without proper planning. This type of design features dense and random deployment of nodes. This will lead to uncovered areas of deployment in the field, which may in-turn need monitoring. Since the nodes are not carefully placed, node failure detection and connectivity management become difficult to handle. Sensor nodes are of different types, which are designed or described based on the type of physical quantity they monitor. As shown in Figure 4, sensors such as humidity, temperature, flow, vibration, power sensors, etc., are connected wirelessly to a base station that is capable of aggregating data gathered from the sensors to the network server. The user computer connects with the server to access the data and further process the data, which will be presented in a format that is more understandable by the decision making team. The wireless media used by these nodes enables a multi-hop communication. These communication links could be provided by radio or infrared technologies. Where a radio link is used, WSN often uses the unlicensed frequency band known as the Industrial, Scientific, and Medical (ISM) band [42]. These frequency bands are specified by the International Table of Frequency Allocations, and they also provide huge spectrum allocation and are unregulated but highly prone to interference.

#### 2.1.3. WSN Topology and Types

The description of WSN topology is associated with the expected capabilities of sensor nodes, which enable the deployment of several numbers of nodes in a sensor field [44]. While four major types of topologies can be adapted for field deployment, such as, star, tree, point-to-point, and mesh topologies [36], well-planned handling of these arrangements are necessary for maintenance purpose [42]. Without a proper topology arrangement, maintenance-related issues become difficult to handle. These issues usually arise in deployment phases: pre-deployment and deployment phase, post-deployment phase, and redeployment of more nodes phase [42]. In some cases, the types of WSN are classified based on the field of application, such as health, industry, military, agriculture, etc. [45]. However, WSN types can be identified as terrestrial, underground, underwater, mobile, and multimedia wireless sensor networks [46].

#### 2.1.4. Challenges Facing WSN

Although WSN technology has had performance improvements over the years [47], with many research questions getting resolved, other challenges are still present. Some of these challenges arise from node structure, while others are posed by the environment in which they operate in. Reference [48] highlighted some of these design challenges encountered by WSN. These include the *operational environments*, where WSN are deployed in areas that affect its operation, such as the industries, underwater, underground, etc.; the *power* consumed by nodes to process its data, transmit it, or receive any data; the *scalability* of these nodes, since they are deployed in large numbers for a specific application; provision of good *quality of service*; *fault tolerance* in the network; limitation in *computational and storage* capabilities; *security* of data in terms of confidentiality, integrity, and availability.

### 2.2. The Internet of Things (IoT) and Industrial Internet of Things (IIoT) Technologies

**Figure 5:**
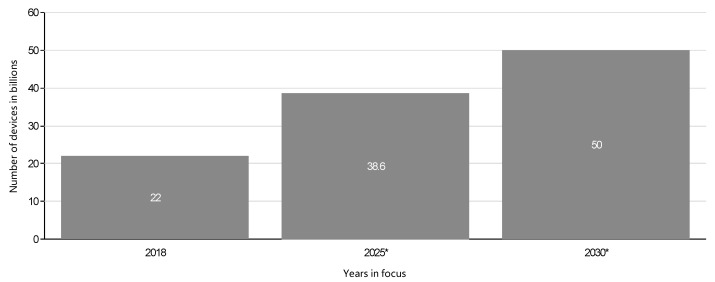
Forecast of the number of IoT-connected devices by 2030 [2].

The advancement in IoT has lessened direct human intervention with objects that are capable of sharing information among themselves and the environment. Designed with sensing and actuating capabilities, these objects have become smart, considering their ability to communicate and autonomously react to real-time or physical events through a unifying infrastructure [49,50]. The interactions of sensors with Radio Frequency Identification (RFID) and the introduction of Internet Protocol (IP), which enables the identification of nodes and their location as well as easy routing on the Internet [51], have transformed WSNs into IoT. Figure 5 shows the forecasted increase in the IoT devices by 2030, however, this is an estimate as signifies by the asterisk. As the number of IoT devices grows tremendously to about 50 billion worldwide [2], the IPv4 identification process will be exhausted, and IPv6 becomes useful for supporting this increase in the IoT paradigm.

Industrial IoT is simply the introduction or application of IoT in industries, especially in manufacturing [20,50]. IoT connectivity is mostly consumer-grade and is used for building automation, human wearables, and messaging, etc., while in IIoT, the connectivity is secured and targeted at the automation of industrial processes such as in defense, aerospace industries, etc. [50]. Table 2 further shows the comparisons between IoT and IIoT based on their respective characteristics [21].

#### 2.2.1. IoT the Enabling Technology for IIoT

**Figure 6:**
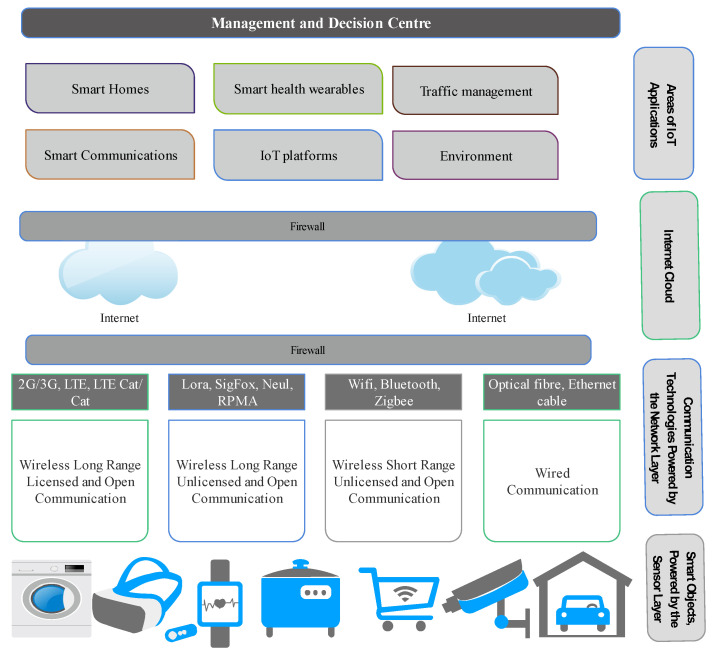
A typical structure of consumer-based IoT showing basic layers.

**Figure 7:**
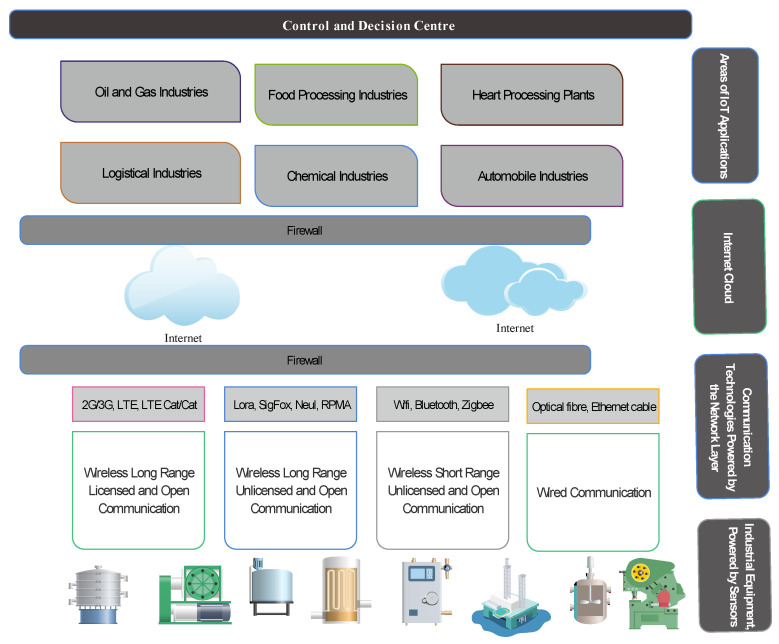
A typical structure of Industrial IoT (IIoT) showing basic layers.

Cloud computing, Machine-to-Machine (M2M), Artificial Intelligent (AI), and Blockchain are among the large group of technologies that enables effective and reliable IIoT [52], with IoT forming the backbone. Figure 6 shows the structure of a typical IoT technology. The *things* here are regular objects, such as a VR headset, watch, electric cooker, parking lot or garage, security cameras, shopping cart, etc., that are made smart because of the sensor nodes embedded in them. This sensing ability enables these things to interact through a common infrastructure, the Internet, with a management center for decision making. The interaction is possible through the various wireless communication technologies available. For the protection of data, a firewall is designed to secure the information exchange between the objects and the center. Communication device manufacturers have provided solutions to protect this data. The network layer provides the platform on which different communication technologies can function. These include long-range licensed and unlicensed wireless bands. The licensed wireless technology, such as 2G/3G and LTE involves, the use of existing telecommunication infrastructures, while the unlicensed wireless (LoRa, ZigBee, etc.) is an ad hoc setup with Internet capability (through an Internet modem or a gateway). The smart objects, set up in this fashion, have various applications such as in homes, city environment, traffic, etc. Their management is also done through any internet-empowered device such as smartphones, laptops, tablets, etc. Figure 7 represents the application of IoT in the industrial environment, the IIoT. In industrial applications, the sensor nodes are embedded in the industrial equipment for monitoring, operations, and management. The communication technologies remain the same; however, the processes that are managed are of critical nature. Applications include oil and gas industries, chemical, automobile, food processing industries, etc. While IoT could be less stringent in terms of criticality, IIoT is critical with timing, reliability, privacy, and security of data being gathered in operation or monitoring.

#### 2.2.2. The IoT/IIoT Communication Technologies

IoT and IIoT are thought to have three or five architectural functionality layers [53,54]. Meanwhile, in [55], for a Man Like Nervous (MLN) system, other layers are involved. Three-layered IoT/IIoT architecture include perception, network, and application layers. While in five-layered architecture, in addition to the three layers are processing and business layers. However, in any type of architecture proposed or adopted, the network or transport [53] layer is responsible for the communication of devices among themselves and with the network infrastructure (e.g., gateway, server, etc.). IIoT allows the industries to meet their operational targets, among other things. However, most of the peculiar needs of the industries are centered on the capabilities of communication technologies as supported by the network layer. These designs need to include *latency*—time required for data propagation and processing, *topology*–device connection style, *throughput*—the volume of data that the network can support, *scalability*—number of supported devices in the network, the *security of data* and *energy*—IoT device operational time without power supply [56]. Table 3 lists the common types of communication technologies used by IoT/IIoT and the network-supported features.

The network or transport layer handles the coverage range of IoT/IIoT. Each type of communication technology has an approximate range it can cover. Therefore, depending on the requirements needed for an application, appropriate technology can be employed. However, for IIoT where timing is critical, latency is an important feature that needs to be put into consideration to achieve reasonable reliability of deployment. To this end, a true picture or understanding of the industrial wireless communication for the application of IoT is necessary.

#### 2.2.3. IIoT Challenges

Some of the challenges faced by Industrial IoT are the requirements needed for their deployment in various settings. Although different fields of application have their requirements [21], most of these requirements are expected or provided by the network layer of the IoT architecture. For example, in [66], the scalability, throughput, latency, energy consumption, topology, and security of data were explained. More so, the interoperability of devices to support the heterogeneous nature of IoT is presented as a future requirement for IoT in [67]. Standardization, architecture, and the need for an increase in storage are some other challenges that IIoT faces [68]. This research paper presents the challenges that are related to the network layer.

*Scalability*: The capacity provided by the global information infrastructure to connect a large number of common objects describes the scalability. This challenge can present itself on different levels [69]. The addressing and identification of devices pose a challenge; however, this issue is mitigated by the use of IPv6 and 6LoWPAN protocols [70]. Due to the large size of these connected objects, a high volume of data communication (big data) and networking capabilities are issues to be tackled. The application layer also contributes to the challenge posed by scalability as a result of the numerous services and service execution options that are generated by heterogeneous connected IoT devices. Therefore, one of the challenges in scalability is the ease of adding new devices to the network without major interruption.

*Latency*: Since the industrial application of IoT is time-critical and usually deployed in environments that are noisy and hard on the wireless communications, the Quality of Service (QoS) provided by IIoT is often characterized by the real-time achievement of the deadline set by an end-to-end communication task, from sensing to control execution in the system [71]. In industries, various communication traffic is generated, and depending on the type, the latency and reliability provided by IIoT can either be least critical or most critical. These types of traffic are explained in [72]. Table 4 presents a summary of the latency and reliability levels needed for the communication traffic.

Industrial traffic (data) that requires fractions of seconds in latency in IIoT communications includes the emergency, critical control, and remote control traffics. This traffic is used for safety purposes, such as the leakage of radiation or poisonous gases in industries [72], continual flow of industrial processes such as automation processes, which require 1 ms latency rate [73], and control of unmanned vehicles, which require 50 ms latency and 1–$10^{-8}$ reliability [74].

*Energy consumption*: The primary energy supply to IoT is batteries. The replacement of these batteries becomes difficult when they are fully drained. However, energy harvesting techniques are becoming promising in resolving this challenge. Ideas and research are already moving toward techniques where solar panels or piezoelectric material will be used to relieve IoT devices from the shackles of battery operations [69]. Meanwhile, efforts have been made to optimize the network protocols in the wireless communication systems. These protocols are optimized to help in reducing the amount of energy consumed by these devices. Examples are the implementation of idle time of the devices when transmission or reception of data is not taking place.

*Security*: Data generated by IIoT are important assets to the industry management, and while lack of proper security of these data is yet to be provided, this provides the reason why some industries are yet to fully deploy IoT. Although some traditional security mechanisms are in use, these are not sufficient enough to protect the complexity of the IIoT systems. These mechanisms include secure protocols in [75], privacy assurance in [76], and lightweight cryptography, as described in [77].

### 2.3. The Standardization and Technology of NB-IoT

NB-IoT is one of the licensed frequency band communication technologies that were standardized by the 3GPP [1]. This technology is fused into LTE infrastructure as it is also an LPWAN-based technology. Among its category of LPWAN but utilizing the unlicensed frequency bands are LoRaWAN, Ingenu, SigFox, etc. NB-IoT is characterized by low-cost deployment and long-range coverage [17]. The use of the already existing cellular infrastructure by NB-IoT makes it a good candidate for the deployment of IoT as it provides a standardized common platform for the connectivity of objects. Among the advantages of NB-IoT include support for an effective cellular communication network, wide radius coverage with bidirectional triggering between data and signaling planes, low power consumption rate, and capacity to support the massive connections of devices [15,78].

#### 2.3.1. Standardization of NB-IoT

**Figure 8:**
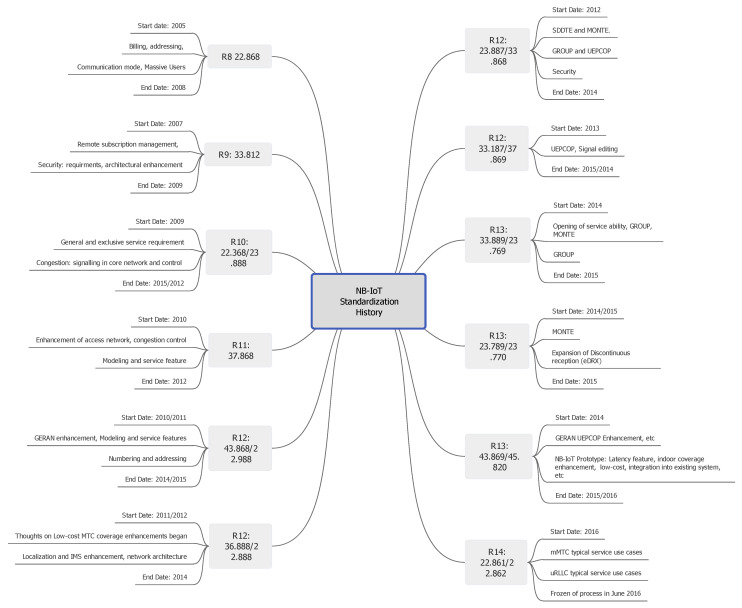
A unique representation of the NB-IoT standardization process.

NB-IoT has passed through a series of standardization by 3GPP. Figure 8 describes a brief history of NB-IoT standardization from inception to its current freezing state. The diagram is a unique representation of the standardization history as it captures the various stages at a single glance. The standardization started in 2005 when 3GPP Release 8 (R8) was specified to cater for massive device connections, billing of usage, addressing, machine type communication mode, and security issues. All the standardization versions have a start date and end date with overlapping of some specific technological fields of concern. Examples are the versions R12 and R13 with various standardization numbers and specific areas of concern. In 2015, Version R13 was released with features that further reduced the energy consumption rate of NB-IoT through the implementation of eDRX and PSM mechanisms [15].

**Figure 9:**
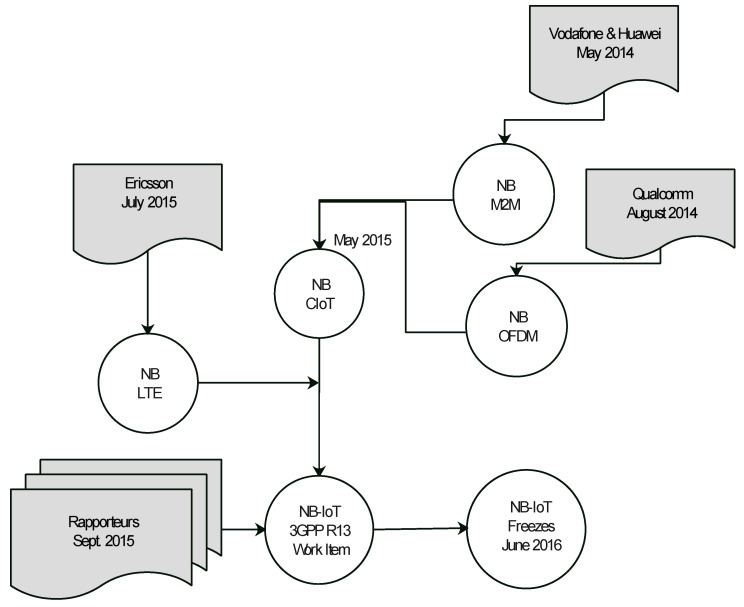
Participating companies in NB-IoT standardization.

Figure 9 shows the major technology companies involved in the standardization process [79]. These companies include Vodafone and Huawei; they paired together to work on NB-IoT M2M communication features and made their submission in May 2014; Qualcomm presented NB-IoT Orthogonal Frequency-Division Multiplexing (OFDM) in August 2014. The works of the above formed the NB Cloud IoT (NB CIoT); a year later, Ericsson presented the LTE features of NB. These works were fused by rapporteurs in September 2015 to form the NB-IoT 3GPP Version R13 work item with a standardization number of 45.820. In this version, the following technological features were addressed: reduced latency, improved indoor coverage, ability to be compatible with existing infrastructure (LTE), support for a low data and low cost terminal.

#### 2.3.2. NB-IoT and Its Features

Some of the main features of NB-IoT are enhanced coverage range, low energy consumption rate meaning long battery life, capacity to connect a massive number of devices per cell, increased reliability, low cost of the terminal and various deployment modes. These features are briefly explained below.

*Coverage and Latency*: NB-IoT gives better coverage as compared to the legacy LTE with 20dB performance. This allows it to deliver to areas that are difficult to reach, such as basements, making it suitable for use in underground car parks. NB-IoT can achieve this coverage either by in-band, stand-alone, or guard band deployment modes. The 20 dB coverage enhancement is supported by retransmission and low-frequency modulation mechanisms. With the two retransmission tones (3.7 and 15 kHz) available for use, NB-IoT can retransmit using up to 128 bits for uplink and 2048 bits for downlink. The latency budget of NB-IoT is 10 s, but a lower latency of 6 s can be achieved for maximal coupling losses as simulated and specified in TR45.820 [1,17].

**Figure 10:**
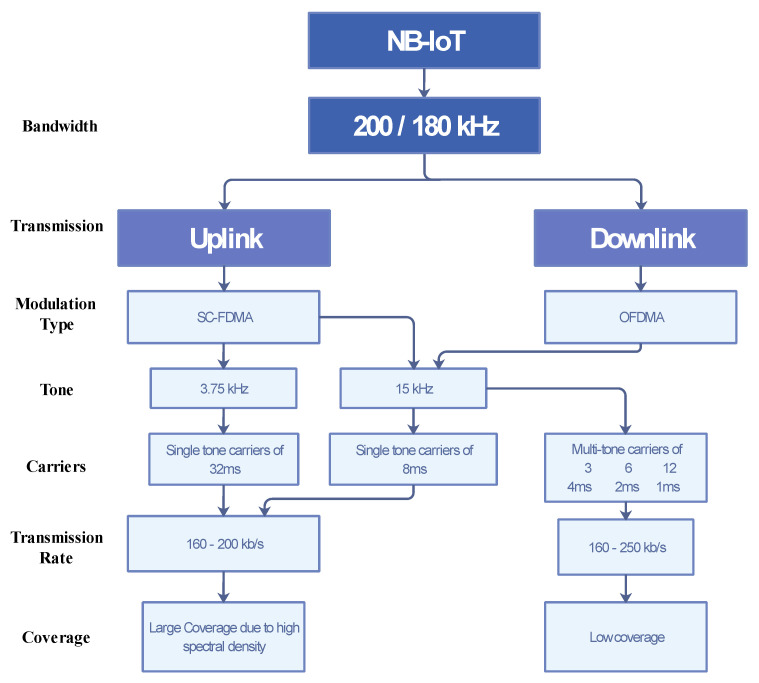
Block diagram of transmission tone mechanism.

The transmission tone feature, which enhances the coverage capability of NB-IoT is presented uniquely in a block diagram in Figure 10. The diagram presents the operational bandwidths of NB-IoT; 200 kHz for stand-alone deployment and 180 kHz for both in-bound and guard-band deployments. For the uplink transmission, NB-IoT uses a Single Carrier Frequency-Division Multiplexing Access (SC-FDMA) modulation scheme. With this modulation scheme, it operates at 3.75 or 15 kHz sub-carrier intervals. These single-tone carriers are at 32 and 8 ms, respectively. The transmission rates used are from 160 to 200 kb/s to achieve a large coverage range that is powered by its high spectral density. The downlink transmission, however, uses an OFDMA modulation scheme with only a 15 kHz sub-carrier interval. It also utilizes multi-tone carriers of 3, 6, and 12 ms that support 160 to 250 kb/s transmission rates. The coverage enhancement of NB-IoT to achieve a 20dB was evaluated in [80], and it was shown that this is true as compared to the legacy LTE.

**Figure 11:**
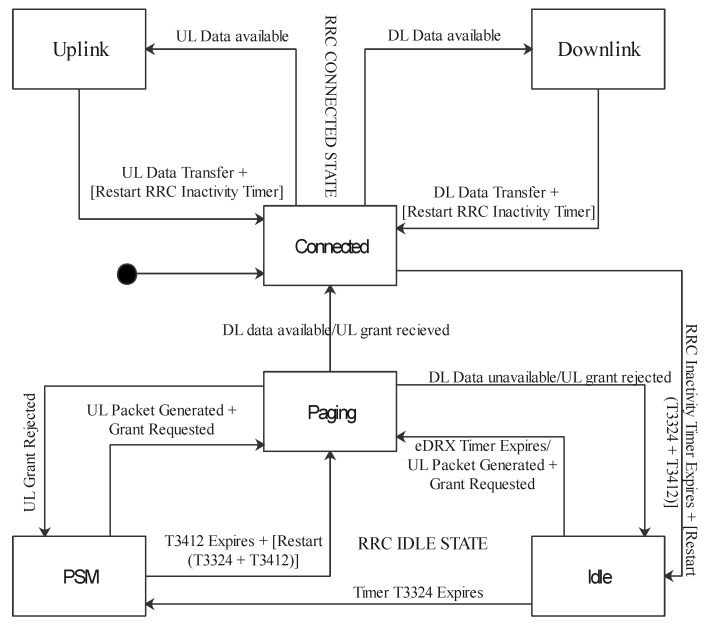
The PSM and eDRX modes.

*Low energy consumption*: The standardization process of the NB-IoT introduced the PSM and eDRX mechanisms for low energy consumption. In the PSM, the device remains registered to the network of transmission but usually enters a deep sleep mode and completely switches off most of its circuitry; at this stage, the device is not reachable from the network. Meanwhile, it could wake up at any time to transmit data when necessary. The eDRX is a temporary idle state that does not listen to the radio channel but periodically becomes active to receive paging messages from the network for possible incoming data before switching to PSM. This periodic activeness is guided by a specified timer, and the paging system is the process of monitoring the control channel for downlink or uplink data indication. Without any activity on the control channel and the expiration of the specified timer, the device switches from the idle state to the PSM state. These two basic features allow for the energy saving of NB-IoT, which surpasses the legacy LTE. Figure 11 shows the idle and paging states [13]. Timer T3324 is the time required to enter into deep sleep mode.

**Figure 12:**
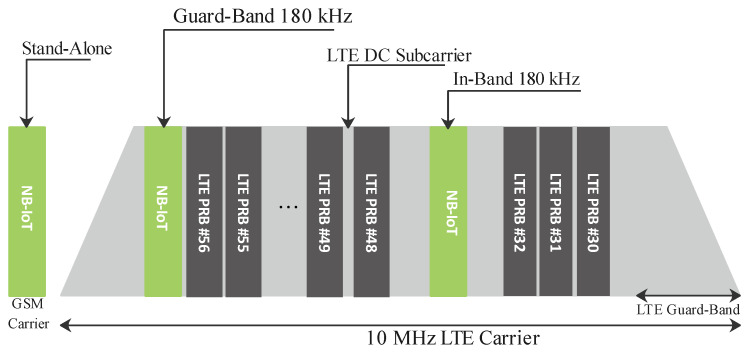
NB-IoT modes of deployment.

*NB-IoT Deployment modes*: NB-IoT is stipulated by RP-151621 to be deployed in FDD transmission [15] and has three types of deployment modes. These include the stand-alone, in-band, and guard-band modes. Figure 12 presents these modes. The stand-alone is an independent type of deployment that utilizes a bandwidth of 200 kHz and does not overlap with the LTE frequency band. The guard-band is a deployment type that utilizes the guard frequency of the LTE or the edge band. It has a 180 kHz bandwidth allocation. Lastly, the in-band deployment is also assigned a bandwidth of 180 kHz and utilizes one of the LTE frequency bands.

#### 2.3.3. NB-IoT Comparison with Other IoT Technologies

In Table 5 [1,7,16,18], the comparison between NB-IoT, other licensed, and unlicensed band IoT is presented. The IoT operating in the licensed bands include eMTC, while LoRaWAN, SigFox, Ingenu, etc., are for the unlicensed band.

Comparisons between NB-IoT and other LPWAN have been carried out to evaluate some features. These features include the cost of deployment, coverage range, interference immunity, power, and spectral efficiencies [6,7,16,81,82]. However, the results obtained showed that these technologies have some distinctive features that are suitable for specific purposes or applications. In some cases, trade-offs are required by the user for their deployments. An example is the deployment of NB-IoT in industries. Such decisions would leverage the existing infrastructure, security, and coverage of the LTE. Meanwhile, a decision that chooses LoRa, would require additional network infrastructure for deployment.

#### 2.3.4. NB-IoT Applications in Industries

**Figure 13:**
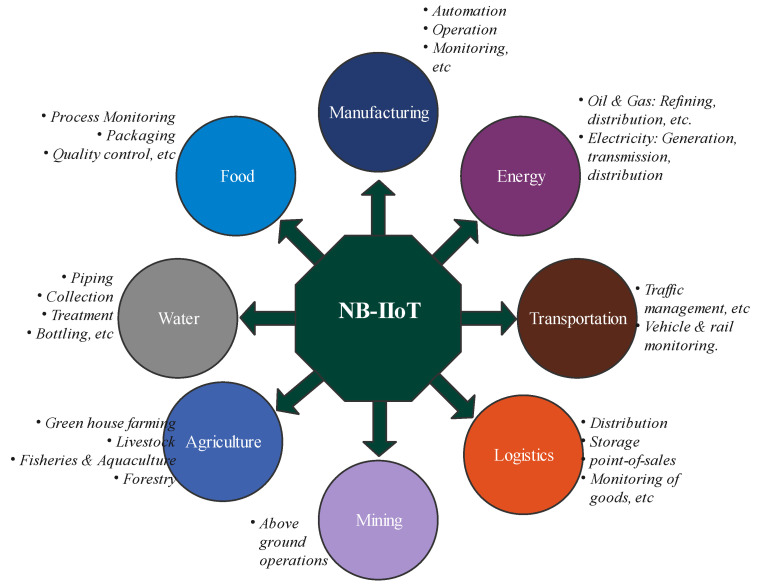
Areas of NB-IoT applications in industries.

The application of NB-IIoT spans many of the industrial sectors with varying degrees of deployments [20]. This usually depends on the criticality and level of confidence in NB-IoT technology. However, its application has been forecast to increase in the foreseeable future especially with the increase in the manufacturing of NB-IoT modules by various vendors [2]. NB-IoT is deployed for different purposes in industries, as shown in Figure 13. In the oil and gas industries, NB-IoT deployment includes refining, distribution, and monitoring of products. Applications are also found in food processing, agriculture, and water industries.

## 3. State-of-the-Art

With the emphasis and success of private mobile networks in industrial settings, the NB-IoT has found its application in the industrial world—Narrow-Band Industrial Internet of Things (NB-IIoT). Its application relies on the existing infrastructure of the legacy LTE. However, the ad hoc WSN forms the bedrock of the IoT. This is because the introduction of a unifying infrastructure that simply allows the identification, addressing and communications of sensors and objects has enhanced the massive connectivity of these devices. This section is organized into two main sections. The first section is about the developmental trends and latest technological features of WSN, IoT, and NB-IoT. The second section presents the application case studies of WSN, IoT, and NB-IoT in industrial environments, respectively.

### 3.1. Developmental Trends and Technical Features

#### 3.1.1. The Wireless Sensor Networks

The observation or monitoring of physical quantities has become necessary over the years. This is not only to provide useful information about the observed entity but also to enable quality decisions. These physical quantities are usually monitored by sensors or nodes. WSN gathers data from the point of observation through a storage unit to the point where decisions are made [36]. The types, applications, and topology of WSN have been described in [35]. The authors in [37] described how WSN can provide the functional requirements in monitoring the environment, which also brings about convenience and efficient methods as compared to the use of human capital.

#### 3.1.2. Internet of Things (IoT)

A commonly acceptable consideration of IoT is where regular objects (physical and virtual, which can be identified either through their physical or virtual attributes) are connected to a scalable and unifying network infrastructure—the Internet. In other words, an infrastructure that can use some sets of regulatory communication protocols for self-configuration to provide a reliable and easy connection for these objects [27]. While the term “Internet of Things” was coined by Kevin Ashton in the year 1999 [28], the basic concept behind it was the combination and application of various technologies such as sensors, Radio Frequency Identification (RFID), and actuators, etc., to bring about the interaction among objects that applies these techniques in a standardized form [29]. These objects are called smart objects because of their ability to interact with the environment and other objects as well.

A survey of some factors and technologies that drives IoT was discussed in [30,31], factors such as communication links (e.g., ZigBee, Bluetooth), identification of objects through electronic product code (EPC) or ubiquitous codes (ucode), smart sensing and computational processes, among other things. The ubiquity of IoT is leading to a paradigm of its kind such as the Internet of People (IoP), Internet of Services (IoS), Internet of Content (IoC), and Internet of Agents (IoA) [32]. Based on the relation of this paradigm with IoT, the authors analyzed these trends and the challenges ahead. The authors in [33,34] reviewed the architecture, networks, and smart objects that form the building blocks of IoT.

#### 3.1.3. Industrial Internet of Things (IIoT)

The dynamic application of IoT to every aspect of human activities has led to its extension in the industrial sector. This extension can be viewed as the development of IoT for the specific application in industries [22,23]. These IoT are expected to meet the unique needs of these industries. The IIoT is considered to be a subcategory of IoT that focuses on industrial communication technologies, M2M applications, etc., as explained in [21]. As a subset, the paper presented the similarities between IoT and IIoT. More so, Reference [20] sees IIoT as a technology that is used to achieve the goals of industries through the deployment of some IoT technologies in a specific way that involves the use of intelligent objects on cyber-physical platforms.

The peculiarity of industrial needs for the application of IoT, among other things, includes quality of service (QoS) [24], and the authors proposed a two-layered architectural distribution of a hybrid IIoT for QoS. One of the aims of IIoT is to provide real-time information to decision-makers about the state of the industry. To achieve this, Reference [25] developed a wireless gateway for the application of IIoT. A framework for the use of Machine Learning (ML) in the processing of generated data from IIoT has been studied in [26].

#### 3.1.4. Narrow-Band IoT, IIoT (NB-IoT, NB-IIoT)

Unlike WSN, IWSN, IoT, and IIoT, the Narrow Band (NB) NB-IoT and NB-IIoT are devices that operate in licensed frequency bands. Following the Third Generation Partnership Project (3GPP), the NB-IoT has a history of standardization from 2005 when version R8 was released to 2016 with the release of version R14 [1]. Among the needs that necessitated the design of NB-IoT is the support for large numbers of IoT devices, extended coverage range due to high spectral density, low power consumption rate, low latency and reliability [1,8]. The coverage range of NB-IoT has been compared with other LPWAN technologies and found to reach 1 km in urban and up to 10 km in rural settlements with a low interference rate [6,7].

NB-IoT has provided requirements to support a large number of devices with a low data rate demanded by industrial operations [5]. The reliability and QoS of NB-IoT is one of the requirements and needs of the industries [9]. NB-IoT also provides ease of deployment to industries, especially in Europe where the network is more established [6].

### 3.2. Application Case Studies in Industrial Environments

#### 3.2.1. Industrial Applications of Wireless Sensor Networks (IWSN)

The application of WSN in the oil and gas industries was presented in [38,39]. The authors discussed the implementation of WSN in the three major sectors of the oil and gas industries. This includes the challenges and requirements for the deployment of WSN. While WSNs are still faced with real-time reliability issues and security, Reference [40] designed and implemented a low-powered ZigBee technology used to monitor and provide a predictive maintenance routine in the oil and gas industry. An example of Industrial Wireless Sensor Network (IWSN) Automation was described in [41]. The performance metrics required for IWSN have necessitated its development as compared to the already existing technologies such as cellular communications, Wi-Fi, etc. These metrics include Low power consumption, the distance between communication points, security, service reliability, data transmission rate, and latency.

#### 3.2.2. Applications of Internet of Things (IoT)

Reference [83] Surveyed the applications of IoT and mobile phone computing and categorized them based on areas of application. The authors’ survey indicates a wide range of applications of IoT in the current state of technology. The applications of IoT also extend to the underground such as mining of mineral resources. In [84], the paper introduced an underground emergency hedging subsystem that aids the use of IoT technology to improve its underground application. In the agricultural field, IoT has found application in arable farming as well, which is detailed in [85]. The paper analytically reviewed the current state of IoT application in arable farming as well as the potential. The authors in [86] explained how the applications of IoT are making things smart, such as smart infrastructure, smart homes, smart traffics systems, smart healthcare, etc. The application of IoT can also be found in an electric power system where the stability of the grid can be improved using an interline power flow controller (IPFC) [87]. The authors explained how this was achieved logically. With the recent emergence and deployment of 5G technology around the world, Reference [88] provides an overview of how IoT could be integrated into the 5G wireless system.

The opportunities provided by IIoT through remote management are one of the main advantages the industries (manufacturers, health services, agriculture, etc.) are leveraging to improve efficiency and productivity [21]. IIoT finds its application in smart manufacturing, for effective wireless communication among IIoT devices and gateways, for example, Reference [89] proposed a Hierarchical Trustful Resource Assignment (HTRA) and Trust Computing Algorithm (TCA) to achieve this, while Reference [90] added a suite consisting of macro-services-based. To provide an effective predictive maintenance culture for Industrial types of machinery, IIoT has been deployed as a solution through the NGS-PlantOne System as detailed in [91]. The presentation of a concept for the application of IIoT in shipyards has been provided in [92]. The connection between physical devices and communication between information systems was studied.

#### 3.2.3. NB-IoT Industrial Application (NB-IIoT)

NB-IoT has been used in Power Wireless Private Networks (PWPN) in power industries [11] to achieve deep and large coverage. The analysis made by the authors also shows that NB-IoT is suitable for the latency tolerance services required in PWPN. To improve the efficiency of next-generation aircraft industries, the low power consumption feature of NB-IoT has enabled its usage in aircraft industries as authored by [93]. The integration of NB-IoT into LTE functionalities has enabled this technology to co-exist with the LTE infrastructure, and its application in smart cities is encouraging [19]. More applications of NB-IoT are found in e-Health [94] and utility industries such as smart metering and tracking [95]. The suitability of NB-IoT deployment in smart energy distribution networks has been tested and optimized in [96].

### 3.3. Challenges in Industrial Environments

#### 3.3.1. WSN in Industrial Settings

The industrial environment has always been harsh to wireless channels. The effect of this phenomenon is visible in the erroneous packet transfer in IWSN. To solve this effect, Reference [97] developed a novel Mixed Integer Programming (MIP) Framework that improves the quality of packet transfer for more battery sustainability in IWSN. The influence on the communication channel of WSN depends on the environment in which they are deployed. This applies to industrial environments as well, and the extent of influence on the channel also depends on the type of industrial setup. In [98], some challenges, such as security, routing of data, etc., were studied in addition to application types of WSN. Reference [99] discussed most of the factors that should be considered when planning WSN deployment for targeted usage. These factors include the communication range of nodes, sensing range, deployment strategy, etc. Reference [45] primarily focused on the challenge of coverage in WSNs. Among the three types of coverage (barrier, point, and area) presented, the authors discussed in detail the coverage area type. The details of which are on typical research work, classified area coverage based on the type of node and deployment, coverage detection, sensing, and communication range.

#### 3.3.2. IoT and Its Challenges

The transformation of IoT has made its application in various fields seamless to some extent. While IoT has made fair progress, this was not possible without some existing and emerging challenges. Some IoT challenges are highlighted in [100] as it affects energy sustainability. The challenges facing IoT range from standardization, scalability, architecture to security, which were highlighted in [68]. Security challenges were the major aspect that Reference [101] focused on. In the paper, based on IoT layers, the authors studied the security issues facing it: requirements, limitations, as well as the way forward. Developing an application for IoT poses challenges for its smooth usage, Reference [102] presented an IoT application development framework (IADev) to mitigate this challenge. The paper described how IADev improves the framework for IoT application improvement.

#### 3.3.3. IIoT Challenges

While the opportunities presented by IIoT cannot be overemphasized, IIoT is still faced with challenges, some of which have received attention in recent years. Reference [21] highlight these challenges. Reference [56] discussed the challenges faced in the application of IoT in logistics industries. These challenges are based on common IoT structural design, which includes network topology, the latency of packets, throughput, power sustainability, the security of data, and scalability of the technology. To solve the latency issue as one of the challenges facing IIoT, Reference [103] proposed a low-latency distributed scheduling function (LDSF). Reliability and latency are important requirements in IIoT for industrial environments. The authors in [104] studied the impact of physical, media access control, and network layers on these two requirements. One of the impacting factors on IIoT in industrial environments is electromagnetic noise. To overcome this, Reference [105] collected electromagnetic noise data from an automobile factory by measuring and characterizing the data for IIoT systems.

#### 3.3.4. NB-IoT in Industries

Areas that are difficult to cover with NB-IoT devices experience bad channel conditions and to mitigate this, rigorous re-transmission or repetition is required. This process requires more energy, which, in turn, reduces the battery life of the devices. More so, there is a restriction regarding large numbers of devices being deployed, which is a result of the unclear performance metrics, such as coverage range, throughput, etc., required in an NB-IoT-supported deployment (guard-band, in-band and standalone) [17]. In [106], the authors analyzed the security issues faced by NB-IoT in a layered manner, which provides substantially different security attack proposals on these layers. NB-IoT technology is applicable in most aspects of an industrial operation; however, to some extent, with wireless deployment, the expectations of factory enhancement fail, especially in the areas of latency, reliability, and resilience. Reference [107] provides problem category classification where the deployment of network technologies is achievable. Generally, intensive interference, high noise levels, and shadowing are some of the factors that make the industrial environment harsh to radio or wireless communications. To overcome these, Reference [108] presented measurements for path-loss and shadowing at 900, 1600, and 2450 MHz to enable proper wireless channel design for industrial setups. The future applications of NB-IoT in health industries as well as the challenges faced are discussed in [109]. To harvest the full potential of next-generation cloud computing, Reference [110] studied the possible challenges that need to be overcome, infusing cloud computing infrastructure, connecting people and devices through IoT.

## 4. Future Research Prospects

Industrial processes undergo several different stages for the final production of goods and services. The continuous efforts in the development and integrations of NB-IoT applications in industries are welcomed in the broader world of IoT. More so, since the involvement of NB-IoT in industrial activities requires a huge number of its deployment for process monitoring and data gathering, many of the solutions provided by vendors are yet to fully satisfy the requirements of the industrial world.

Among the key requirements of industrial settings are: NB-IoT as a Self-Organizing Network, leveraging on edge computing to improve scalability in the deployment of an NB-IoT network, assurance in the security of industrial data generated, and having a suitable NB-IoT model that meets its propagation challenges.

### 4.1. Self-Organizing Network (SON) and NB-IoT Management

The use of NB-IoT in industries has made activities easy, safe, effective, and more productive. However, as the number grows, the management of these devices becomes cumbersome and complex. To achieve a continuous and smooth production level, more physical efforts are required to maintain these devices. While some attempt has been made to establish network management systems (NMS), this form of management still requires physical configuration, registration, and maintenance of devices whenever they breakdown or are introduced into the network system. The management of NB-IoT is not limited to device management where provision for automatic control, including firmware upgrade, activation and deactivation, and monitoring capabilities, is required, but also network management functionalities are required to maintain network topology efficiency, synchronization of devices, and traffic congestion control. Data management can not be left out as this involves the ability to aggregate raw data for data analytics and data recovery [111].

**Figure 14:**
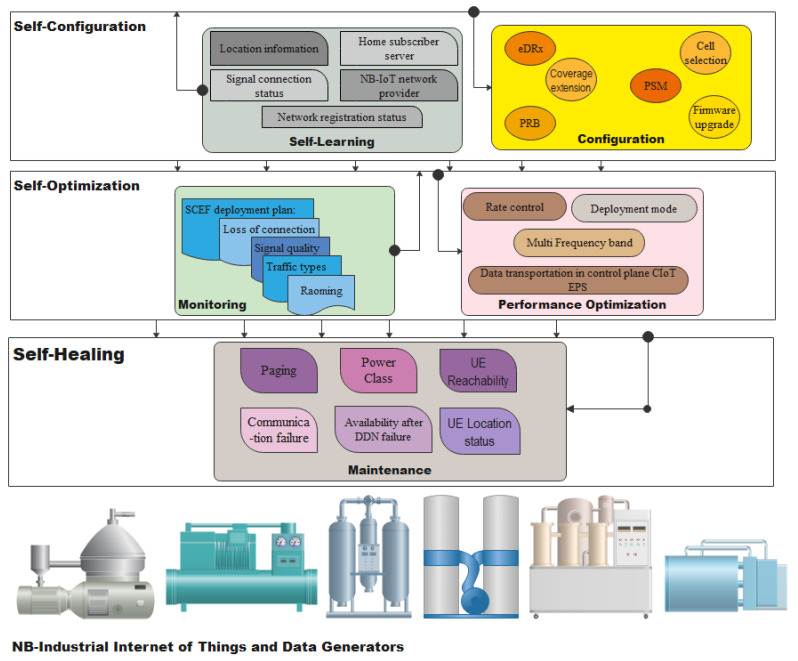
Application of SON in NB-IoT networks.

Since NB-IoT is a superimposition on LTE and its applications are constantly increasing in number, all the main drivers of SON that apply to LTE are also applicable to NB-IoT. These drivers include complexity and a large number of NB-IoT structured network parameters, an increase in the number of base stations (eNB) to which NB-IoT devices are attached, and a requirement of regular human intervention for configurations and management [112]. In Figure 14, the main functionalities of SON are explained as related to NB-IoT applications. The first process involves self-configuration, a process where necessary and basic configurations for system operation are automatically performed for newly deployed modules. This process starts with a stage called self-learning where parameters, such as the NB-IoT network provider, network registration status, location information, home subscriber servers, signal connection status viz idle or connected, and update on NB-IoT PSM, are all learned. This is followed by the configurations of the operational parameters, which include: eDRx, PSM, cell selection, coverage extension, Physical Resource Block (PRB), and firmware upgrade. The second process is the self-optimization process, which involves monitoring of events and performance optimization. NB-IoT uses the Service Capabilities Exposure Function (SCEF) deployment plan to monitor events, and these events include: roaming, loss of connection, traffic types viz critical, remote, and non-critical traffics, and signal quality. The performance optimization stage optimizes data transportation in the control plane Cellular-IoT (CIoT) Evolved Packet System (EPS), rate control for quality assurance, roaming for hand-off, multi-frequency band, and deployment mode for bandwidth. The third process of SON is self-healing, where paging and power class are used for outage and power maintenance. NB-IoT employs SCEF plans for User Equipment (UE) reachability, location, and change in location of the UE, communication failure, availability after DDN failure, and change of IMSI-IMEI (SV) associations [113].

### 4.2. Edge Computing and Scalability in NB-IoT Network

Scalability in an NB-IoT network requires the introduction of new devices into the network without any reduction in the network performance and minimum human intervention. To achieve any level of scalability in the network, some challenges have to be resolved. The interoperability among devices deployed, memory space needed for data storage, the processing power of the system, and bandwidth for smooth and effective data transmission are all parts of the key challenges that need to be resolved for efficient scalability [114].

In order to mitigate some of these challenges, the edge computing technology can be deployed to provide a more robust and cost effective computing power in terms of memory, speed, and enhanced scalability to the NB-IoT network. Edge computing, however, is an intermediary between the NB-IoT network and cloud computing. Therefore, this proposed system will require a stable and reliable cloud computing platform that supports NB-IoT for industrial cases [115]. Resolving these challenges will go a long way to build the confidence of industrial managers in the implementation of NB-IoT networks.

### 4.3. Security of Data in Industrial NB-IoT

Protecting data from unauthorized access and corruption throughout its life cycle is a huge concern to every industrial management team. Therefore, securing NB-IoT data generation in an industrial environment incorporates a complex set of industrial processes and practices that needs to accommodate both reliability and safety requirements [50]. These requirements are not limited to but include the following: confidentiality and integrity of data and users. This can be achieved through a protection policy that allows secured, encrypted, version control, error detection, backup and recovery procedure mechanisms, password management, two-factor authentication, biometric verification, and access control for users. This is because confidentiality and integrity in data allows accurate, complete, and unmodified data to be accessed by only approved users, a requirement that ensures the continued survival of industrial practice. Service availability is a requirement that ensures easy access to NB-IoT data whenever they are needed. To meet this requirement, NB-IoT data must incorporate a mechanism that supports off-sight back-ups or mirroring, a quick and safe data recovery system, and a redundancy mechanism to avoid complete network failover. However, to avoid data breaches and failures, a constant and proper monitoring policy needs to be put in place by industrial management. Data freshness is another requirement paramount to NB-IoT data security, owing to the limitation in the memory capacity of these modules and the need to have up-to-date data for decision making using the generated data. To overcome this challenge, a secured Cloud Internet of Things framework can be deployed to provide this memory space requirement.

### 4.4. Suitable NB-IoT Industrial Propagation Model

Since the industrial wireless environments are characterized by high reflectiveness, which causes multi-path effect and electromagnetic resonance, this phenomenon creates significant harshness on wireless communication in indoor industrial environments [116]. While every industry is unique in its setup, many works of literature have presented propagation models that attempt to adequately describe the characteristics of wireless propagation in industries [3,107]. Understanding the wireless characteristics of an indoor industrial environment in terms of its stringent quality of service (QoS) requirements is necessary and makes it easy to plan and design wireless systems suitable for industrial applications. A comprehensive comparative analysis of channel models among low-powered and high-powered IoT (including NB-IoT) for industrial applications, and a generic propagation path-loss (PL) for 900 MHz application for the indoor industrial environment was presented as shown in Equation (1) [4].

(1) $$PL=61.65+24.9\log_{10}\left(d_{3D}/15\right),\;\sigma =7.35$$

Therefore, to the best of our knowledge in this survey, the wireless propagation of NB-IoT in an indoor industrial environment still requires further research that investigates the latency, reliability, and coverage characteristics of its propagation. Further research in this respect is already ongoing, and subsequent addressing of these challenges will give the industry confidence in the wireless connectivity of their devices and processes.

The success of NB-IoT and the subsequent decentralization of industrial processes will allow rearrangement of the hierarchy of industrial flows, information and processes leading to a more productive environment. This is where we believe state-of-the-art distributed ledger technologies in harmony with edge computing will allow for rich analytical information that could unlock industrial processes that were never even considered before [10]. For instance, in addition to the further directions discussed above, blockchain-based offerings looking to disintermediate the business of outsourcing storage and computing will break the monopoly of expensive, wasteful and environmentally harmful dominance of corporate data-centers, bringing full control of processes to local industries. Research efforts in these areas that decentralize the data, analyze it using machine learning techniques and then use edge computing for real-time analytics will unlock several industrial applications.

## 5. Conclusions

In this work, we presented a review of the Narrow-Band Internet of Things (NB-IoT) and its application in industries (NB-IIoT). The reviews of Wireless Sensor Networks (WSN), as well as IoT, were made. These were presented in a manner that shows the relationships between these technologies. The review, among other things, covers the technological features, applications, and challenges. A novel presentation of the transmission tone mechanism of NB-IoT was made. Comparisons were made between NB-IoT and other Low Power Wide Area Networks (LPWAN). The paradigm presented by NB-IoT allows a brief and unique pictorial overview of its standardization process as provided by 3GPP. While NB-IoT is an integration into the legacy LTE and increases its application in industries, this work presents the wireless communication challenges that the technology faces in the industrial environment. As a future research direction, we present the need for future research into wireless communication of NB-IoT in industrial environments. These include the need for NB-IoT networks to be self-organizing in a manner that meets the industrial requirements. Furthermore, we also discussed the application of edge computing to improve the scalability of the network management, securing the data to maintain confidentiality, integrity, and availability, and finally, providing a suitable propagation model that describes the industrial wireless system.

## Figures and Tables

**Table 1:** Highlights of some research work.

Category	Discussion	Ref	Year
NB-IoT	Survey the features of NB-IoT and its application in industries	[5]	2019
	Features of some LPWAN are compared for industrial applications	[6]	2019
	Overview of NB-IoT	[1,7]	2017
	Evaluates the coverage performance of NB-IoT	[8]	2016
	Presents NB-IoT architecture through the perspective of 3GPP	[9]	2017
	Explanations on the automation of Industries with Narrow-Band Internet of Things Technology	[10]	2020
	Did a comparative analysis between two LPWAN viz; NB-IoT and LoRa on Power Wireless Private Network (PWPN)	[11]	2018
	Studied the performance of NB-IoT and its latency-energy levels.	[12]	2020
	Evaluates the Power Saving Mode (PSM) and Expanded Discontinues Reception (eDRx) schemes of NB-IoT and a developed energy model	[13]	2018
	Surveyed the energy efficiency of NB-IoT, its applications and challenges	[14]	2019
	Highlights the applications and implementations of NB-IoT	[15]	2017
	NB-IoT, SigFox, Lora and GPRS coverage range were compared in an area of 7800 km${}^{2}$	[16]	2017
	A study of the technical representation of NB-IoT from physical to its media access control layers	[17]	2019
	Proposed an uplink adaptation scheme for uplink scheduling in NB-IoT	[18]	2017
	Studied the performance of NB-IoT and eMTC in the implementation of smart cities	[19]	2018
IIoT	Analytically proposes IIoT architecture	[20]	2018
	Presents the opportunities in IIoT, its features, and challenges	[21]	2018
	Review on IIoT	[22,23]	2018
	Optimized QoS through wireless transmission in IIoT	[24]	2020
	Demonstration of IIoT by the inclusion of gateway	[25]	2019
	Uses machine learning for IIoT	[26]	2020
IoT	Identify the evolution of radio frequency identification in IoT technology	[27]	2008
	Survey the impact of IoT on technology, social, and businesses	[28]	2018
	Survey the architecture and features of IoT	[29,30,31,32,33,34]	2018, 2017, 2016, 2020, 2019, 2019
WSN	WSN and its types surveyed	[35]	2018
	Survey WSN in Agriculture	[36]	2016
	Discuss the application of WSN in the monitoring of the environment	[37]	2012
	Presents the requirements and challenges in the application of WSN in oil and gas industries	[38]	2018
	Proposes the use of WSN and IoT in oil and gas industries	[39]	2017
	Described application of WSN in industrial automation	[40]	2008
	The standardization process in industrial WSN for industrial automation	[41]	2010

**Table 2:** Comparisons of IoT and IIoT characteristics.

Types	Data Volume	Connectivity	Exchange of Information	Market Segment	Criticality	Impact
IoT	Big data	Consumer grade, e.g., smart homes, entertainment, etc. Business to consumers, business to business to consumers	Service providers, consumers, limited enterprises, and small businesses	Not stringent (Excluding medical applications)	Revolution	
IIoT	Specific and limited data	Secured, e.g., health care, energy, etc.	Business to business only	Enterprises and Industries	Critical for timing, reliability, privacy, and security	Evolution

**Table 3:** Common IoT/IIoT wireless communication features.

Type	Latency	Bandwidth	Data Rate	Coverage Range	Energy	Security	Scalability	Ref.
NB-IoT	1–10 s	200 KHz	200 kbps	1 km/10 km (Urban/Rural)	10 years battery life	Yes	52,000	[5,6,9,57,58,59]
LoRaWAN	600 ms	250 KHz and 125 KHz	50 kbps	5 km/20 km (Urban/Rural)	>10 years battery life	Yes	Approx. 104 nodes/BS	[5,6,58,59,60]
SigFox	1–20 s	100 Hz	100 bps	10 km/40 km (Urban/Rural)	>10 years battery life	Yes	Approx. 106 nodes/BS	[5,6,58,59,60]
Bluetooth	200–500 ms	1 MHz	3 Mbps	10 m	72 microwatts	Yes	8	[58,59,61,62,63,64]
Wifi	20–250 ms	22 MHz	11 Mbps–10 Gbps	100 m	0.2 watt	Yes	2007	[58,59,61,62,63,64]
ZigBee	60–150 ms	0.3/0.6 MHz, 2 MHz	250 kbps	10–100 m	90 microwatts	Yes	65,000	[58,59,61,62,63,65]

**Table 4:** Latency and reliability summary for industrial communication traffic.

Traffic Types	Areas of Application	Reliability	Latency	Criticality
Emergency traffic	Safety traffic	Ultra-high	Sub-milliseconds	Most
Critical control traffic	Robotic motion control	Ultra-high	Milliseconds	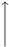
Remote control traffic	Unmanned vehicle control	High	Milliseconds	
Critical Alerting	Periodic control checks	Medium	Seconds	
Non-critical Alerting	Temperature measurement	Low	Minutes	
Monitoring	Static feedback	Low	Minutes	Least

**Table 5:** Comparison between NB-IoT frequency band and other bands.

Parameters	eMTC	NB-IoT	LoRa	SigFox
Spectral Bandwidth	1.4 MHz	180 KHz	7.8–500 KHz	200 KHz
Spectral Frequency Band (MHz)	700–900	700–900	868	868
	Cellular	Cellular	ISM	ISM
	Licensed	Licensed	Unlicensed	Unlicensed
Spectral Efficiency	High	High	Very Low	Very Low
Data Rate	<1 Mbps	<50 kbps	<10 kbps	100 bps
Coverage Area	<10 km	<15 km	<10 km	<12 km
Terminal Cost ($-2020)	3.3	2–3	2.64	2.64
Power Efficiency	Medium-High	Medium-High	Very High	High
Interference Immunity	Medium	Medium	Very High	Medium
Standard	3GPP Release 14	3 GPP Release 13	LoRaWAN2	ETSI

## Data Availability

Not applicable.
